# PRMT5 Promotes Pancreatic Cancer Tumorigenesis via Positive PRMT5/C‐Myc Feedback Loop

**DOI:** 10.1002/mco2.70150

**Published:** 2025-05-15

**Authors:** Fan Yang, Ping Song, Zhaofeng Xiao, Renyi Su, Xin Fang, Yichao Wu, Xiao Xu, Kai Wang

**Affiliations:** ^1^ Department of Vascular Surgery Affiliated Hangzhou First People's Hospital School of Medicine Westlake University Hangzhou China; ^2^ Department of Gastroenterology Affiliated Hangzhou First People's Hospital School of Medicine Westlake University Hangzhou China; ^3^ Key Laboratory of Integrated Traditional Chinese and Western Medicine for Biliary and Pancreatic Diseases of Zhejiang Province Hangzhou China; ^4^ Hangzhou Institute of Digestive Diseases Hangzhou China; ^5^ The Fourth School of Clinical Medicine Zhejiang Chinese Medical University Hangzhou China; ^6^ School of Clinical Medicine Zhejiang University Hangzhou China; ^7^ Department of Hepatobiliary Pancreatic and Minimal Invasive Surgery Zhejiang Provincial People's Hospital (Affiliated People's Hospital) Hangzhou Medical College Hangzhou China; ^8^ NHC Key Laboratory of Combined Multi‐Organ Transplantation Hangzhou China; ^9^ School of Clinical Medicine Hangzhou Medical College Hangzhou China

**Keywords:** pancreatic cancer, PRMT5, c‐Myc, positive feedback loop, proliferation, therapeutic targets

## Abstract

The oncogenic role and underlying mechanism of PRMT5 in pancreatic ductal adenocarcinoma (PAAD) remained to be elucidated. In this study, we aimed to investigate the oncogenic role, underlying molecular mechanisms, and potential therapeutic value of PRMT5 in PAAD. PRMT5 was significantly upregulated in pancreatic cancer than adjacent nontumor pancreas, which was positively correlated with poor prognosis. Genetic and pharmacological inhibition of PRMT5 suppressed PAAD proliferation in vitro and in vivo, exhibiting promising therapeutic effect in vivo. Mechanistically, PRMT5 directly bound to the promoter region of c‐Myc and activated its transcription. Transcriptionally activated c‐Myc in turn inhibited proteasome‐mediated degradation of PRMT5 and enhanced its protein stability, resulting in increased PRMT5 expression. The maintained PRMT5 further enhanced the transcription of c‐Myc. In conclusion, PRMT5 forms a positive feedback loop with c‐Myc to promote the proliferation of pancreatic cancer. Targeting this oncogenic communication may represent a novel and potential therapeutic approach for pancreatic cancer.

## Graphical abstract

By forming a positive feedback loop with c‐Myc, PRMT5 promoted the pancreatic cancer tumorigenesis. Inactivation of PRMT5 with small molecular inhibitors led to suppressed proliferation, colony formation, enhanced apoptosis, and cell cycle arrest. Targeting PRMT5 exhibited promising therapeutic effect for pancreatic cancer

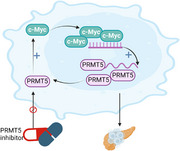

## 1 Introduction

Pancreatic cancer is a lethal malignancy, whose 5‐year survival rate is less than 7% [1]. As a malignancy of high proliferation and metastasis capability, the majority of the pancreatic cancer are initially diagnosed at advanced stage and not suitable for surgery. For those who receives surgical resection, high recurrence severely limits the prognosis. Therefore, it is urgent to get a more comprehensive view of the molecular mechanism underlying the malignant biology of pancreatic cancer.

Increasing attention has been put on posttranslational modifications (PTMs) such as arginine methylation. Protein arginine methylation is involved in diverse cellular processes, including proliferation, transcription activation, protein degradation, and so on. Protein arginine methyltransferase (PRMTs) catalyzed arginine methylation [2, 3, 4]. According to the catalytic type, PRMTs are classified into three categories: (1) type I (PRMT1, 2, 3, 4, 6, and 8) catalyzes both asymmetrical dimethylation and monomethylation of arginine (MMA); (2) type II (PRMT5 and 9) catalyzes symmetrical demethylation (SDMA) and MMA; and (3) type III (PRMT7) exclusively catalyzes MMA [5]. Recently, increasing studies have reported that high PRMT5 expression and its correlation with poor prognosis in multiple malignancies [6, 7, 8, 9, 10], including pancreatic cancer [11].

PRMT5 plays an oncogenic role in pancreatic cancer in various ways. Sun et al. [12] reported that PRMT5‐mediated methylation suppressed the autophosphorylation and kinase activity of the Hippo pathway initiator MST2, leading to inactivation of the Hippo signaling axis and promoting progression in pancreatic cancer. PRMT5 promoted the epithelial–mesenchymal transition (EMT), promoting migration and invasion in pancreatic cancer [13]. PRMT5 inhibition exhibited synergistically enhanced cytotoxicity of other chemotherapeutic agents in pancreatic cancer. Wei et al. [14] reported that PRMT5 inhibition led to the accumulation of excessive DNA damage, RPA depletion and impaired homology‐directed DNA repair activity. Combination of PRMT5 inhibition with gemcitabine resulted in conditional lethality and a synergistic reduction in pancreatic cancer growth. PRMT5 inhibition also improved sensitivity to palbociclib in pancreatic cancer [15]. Qin et al. [16] reported that PRMT5 epigenetically silenced F‐box/WD repeat‐containing protein 7 (FBW7) to stabilize c‐Myc, leading to enhanced proliferation and aerobic glycolysis. Increasing evidence suggest that PRMT5 is a promising therapeutic target for pancreatic cancer, and further research of its functions and underlying mechanisms is of great significance.

Emerging evidence suggested the existence of regulation between PRMT5 and c‐Myc [13, 16, 17, 18]. In this study, we found PRMT5 promoted the proliferation, and both genetic and pharmacological inhibition of PRMT5 impeded pancreatic cancer tumorigenesis. Mechanistically, PRMT5 directly bound to the promoter region of c‐Myc and activated its transcription. Consequently, increased c‐Myc inhibited proteasome‐mediated degradation of PRMT5 and enhanced its protein stability, maintaining PRMT5 expression. Collectively, our study revealed the novel PRMT5/c‐Myc positive feedback loop via which PRMT5 exerted its pro‐proliferation function. Targeting this feedback loop might be a potential therapy for pancreatic cancer.

## 2 Results

### 2.1 PRMT5 is Highly Expressed in Pancreatic Cancer and Correlates with the Poor Prognosis

**FIGURE 1:**
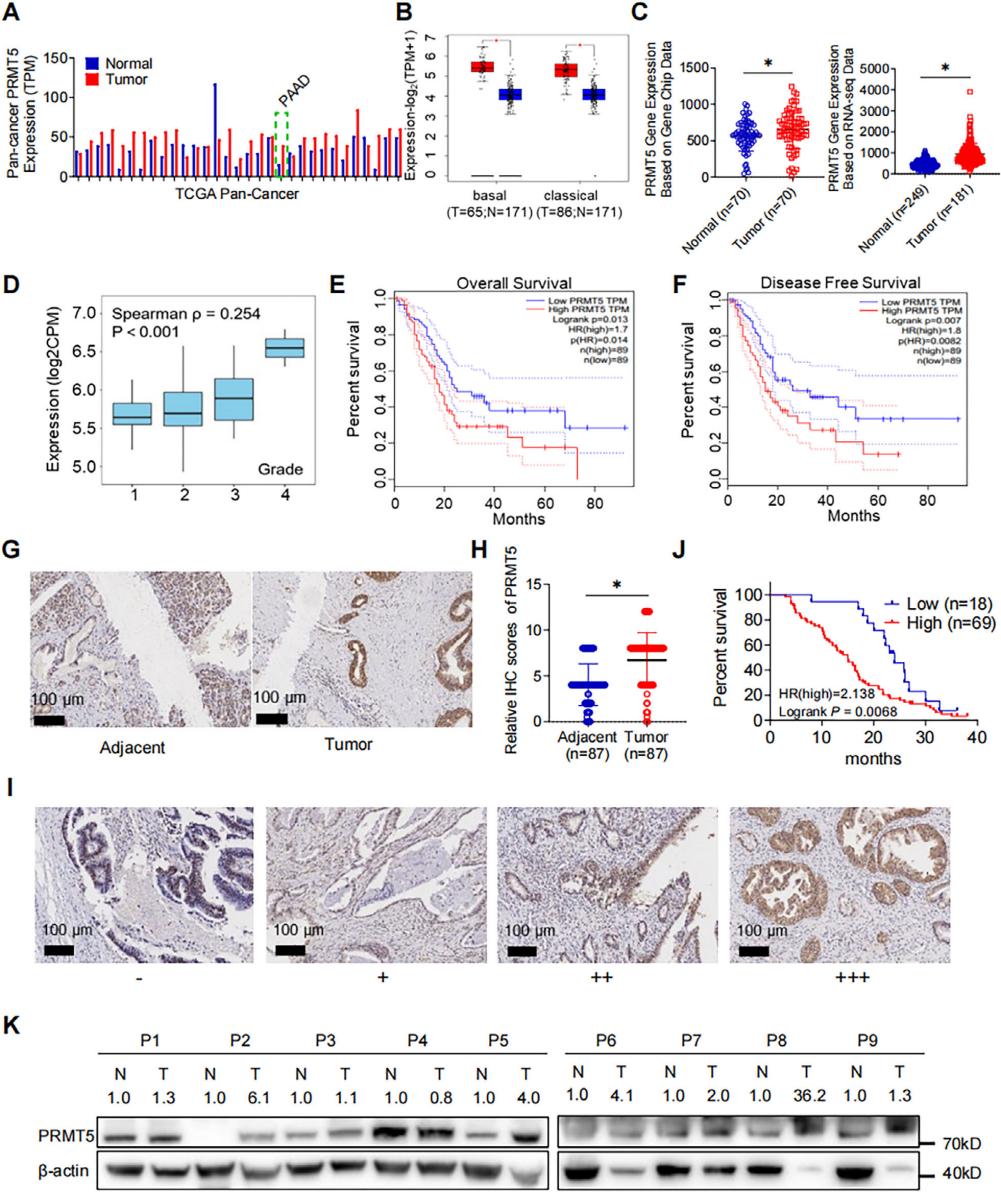
High expression of PRMT5 correlates with a poor prognosis in Pancreatic Cancer. (A) Pan‐cancer analysis of PRMT5 mRNA expression in tumor and adjacent normal tissue based on TCGA datasets. (B) Expression analysis of PRMT5 mRNA expression in both basal and classical subtypes based on TCGA‐PAAD dataset. (C) Gene chip‐based expression analysis of PRMT5 in pancreatic cancer (left), and RNA‐seq based expression analysis of PRMT5 in pancreatic cancer (right). (D) Expression analysis of PRMT5 mRNA in pancreatic cancer of different differentiation grade. (E and F) Kaplan‐Meier survival analysis of patients’ overall survival (OS) and disease‐free survival (DFS) with different PRMT5 mRNA expression. (G) Representative images of IHC staining for PRMT5 in human PAAD tissue array. Scale bar, 100 µm; (*n* = 87). (H) Statistics of PRMT5 IHC score between PAAD and adjacent normal pancreas. (I) Representative IHC staining images of PAAD patients with different PRMT5 protein levels. (J) Kaplan‐Meier survival analysis of patients’ overall survival (OS) with different PRMT5 protein expression, Log‐rank method. (PRMT5 high or low was determined based on the comparison to the median PRMT5 protein expression). (K) Western blot of PRMT5 protein expression in 9 pairs of fresh pancreatic cancer and adjacent non‐tumor pancreas. Unless specifically indicated, bars represent mean ± SD of technical replicates, p value measured by unpaired T‐test and * represent values of < 0.05. PAAD, pancreatic ductal adenocarcinoma; IHC, immunohistochemistry.

Based on TCGA database, we performed pan‐cancer expression analysis of PRMT5 mRNA and found that PRMT5 mRNA was upregulated in tumor tissue than adjacent nontumor tissue in most cancers, including pancreatic cancer (Figure 1A; pancreatic ductal adenocarcinoma [PAAD], the green dash‐line box). Further analysis revealed the same expression pattern in both basal and classical types of PAAD (*p* < 0.05; Figures 1B). A similar pattern was observed in both Gene Chip data and RNA sequence data (Figure 1C). A positive correlation between PRMT5 mRNA level and tumor grade was observed (TISIDB database, *ρ* = 0.254, *p *< 0.01; Figure 1D) [19]. In addition, higher PRMT5 mRNA levels could indicate poorer overall survival (OS) and disease‐free survival (DFS) in PAAD patients (*p* < 0.05; Figures 1E,F and S1A‐D).

We then questioned whether the protein level of PRMT5 was upregulated in PAAD and its potential impact on patients’ prognosis. We analyzed the pancreatic tissue array. Immunohistochemical staining (IHC) staining revealed significantly higher protein level of PRMT5 in PAAD tissues than in adjacent nontumor tissue (Figure 1G,H; *p* < 0.01), which was consistent with public data (Figure S1E; The Human Protein Atlas database) [20]. Next, we investigated whether patients of different PRMT5 protein level exhibited distinct outcome. PAAD patients were categorized as PRMT5 high or low group according to the PRMT5 IHC score (Figure 1I). Clinical characteristics were compared between the two groups. Higher TNM staging, lymph nodes invasion, distal metastasis, and vascular invasion were significantly increased in PRMT5 high group (Table S1). Survival analysis revealed worse OS in those patients with higher PRMT5 score (Figure 1J; Log rank *p *= 0.0244). We then evaluated the expression of PRMT5 in nine pairs of pancreatic cancer and adjacent normal tissue, and the western blot detection exhibited higher PRMT5 in the pancreatic cancer (Figure 1K). Taken together, PRMT5 is upregulated in PAAD, and high expression of PRMT5 indicates poor prognosis.

### 2.2 PRMT5 Inhibition Suppresses Proliferation of PAAD in Vitro

**FIGURE 2:**
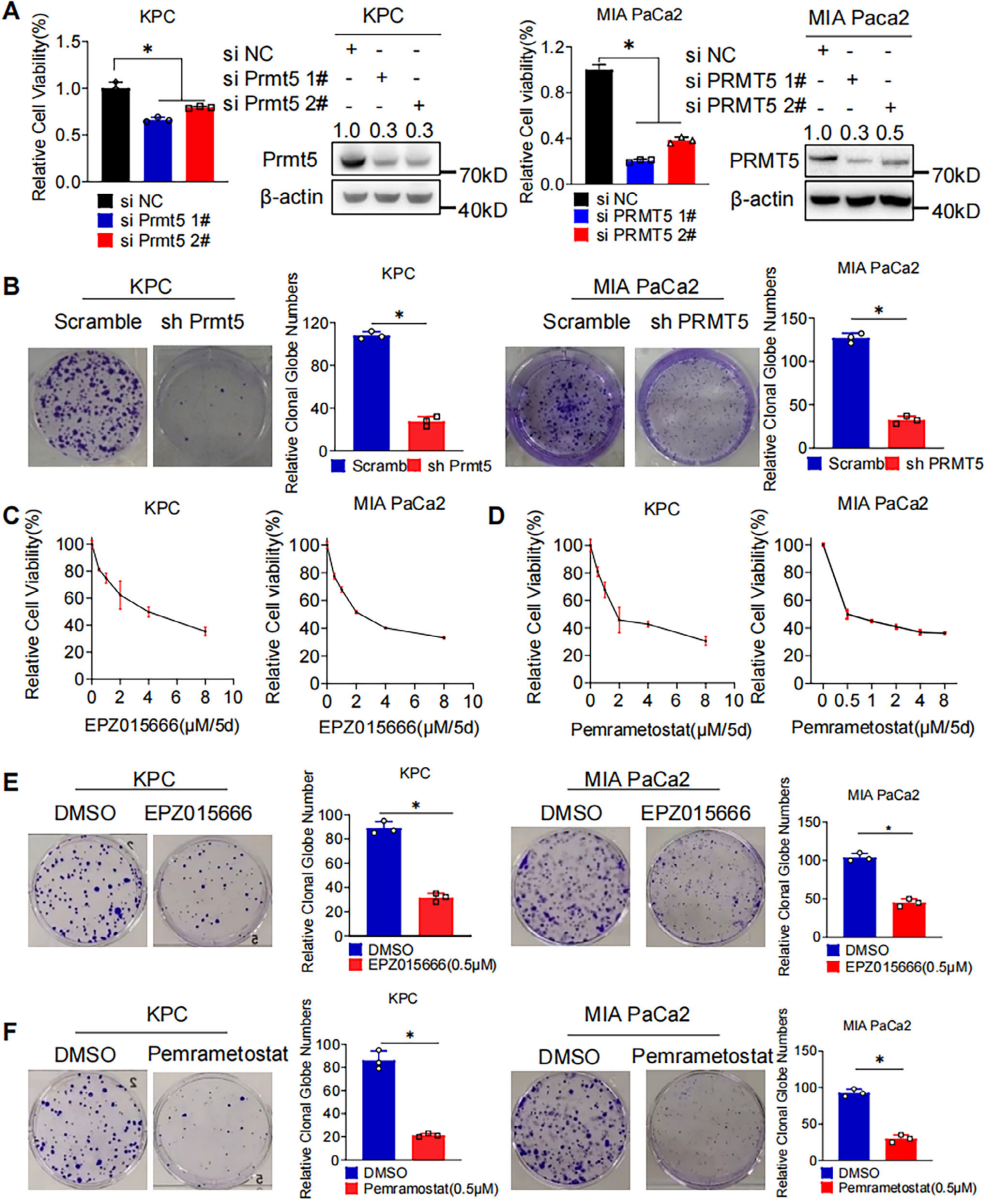
PRMT5 Inhibition Suppresses Proliferation of PAAD Cells in Vitro. (A) Cell viability of PAAD cells (KPC, left. MIA PaCa2, right) with or without genetic PRMT5 inhibition was measured by MTS assay. (B) Colony formation assay of PAAD cells (KPC, left; MIA PaCa2, right) with or without genetic PRMT5 inhibition. (C) Cell viability of PAAD cells (KPC, left. MIA PaCa2, right) after pharmacological inhibition (EPZ015666, 0.5µM to 8µM, 5 days) was measured by MTS assay. (D) Cell viability of PAAD cells (KPC, left. MIA PaCa2, right) pharmacological inhibition (Pemrametostat, 0.5µM to 8µM, 5 days) was measured by MTS assay. (E) Colony formation assay of PAAD cells (KPC, left. MIA PaCa2, right) with or without pharmacological PRMT5 inhibition (EPZ015666, 0.5µM, 14 days). (F) Colony formation assay of PAAD cells (KPC, left; MIA PaCa2, right) with or without pharmacological PRMT5 inhibition (Pemramostat, 0.5µM, 14 days). Unless specifically indicated, bars represent mean ± SD of technical replicates, p value measured by unpaired T‐test and * represent values of < 0.05.

We performed western blot to assess the expression level of PRMT5 protein in normal pancreas cell line and pancreatic cancer cell lines (Figure S2A; normal human pancreatic ductal cell: HPNE. human pancreatic cancer cell lines: AsPC‐1, BxPC‐3, MIA PaCa2, PANC‐1; murine pancreatic cancer line: KPC). KPC, BxPC‐3, and MIA PaCa2 were utilized for further experiments. Genetic inhibition of PRMT5, that is, PRMT5 knockdown with small‐interfering RNAs (siRNA) or shRNA, suppressed cell growth and colony formation in PAAD cells (Figures 2A,B and S2B–D). To confirm whether inhibition of PRMT5 could suppress proliferation in PAAD cells, two kinds of PRMT5 inhibitors EPZ015666 and Pemramostat were administrated to cell culture. Pharmacological inhibition of PRMT5 resulted in dose‐dependent viability suppression and decreased colony formation in PAAD cells (Figures 2C–F and S2E–G). We then performed scratch assay in PAAD cells with or without PRMT5 inhibition. Similarly, PRMT5 inhibition led to suppressed healing (Figure S2H–I). In general, PRMT5 promotes the proliferation of PAAD.

### 2.3 PRMT5 Inhibition Induces Apoptosis of PAAD Cells in Vitro

**FIGURE 3:**
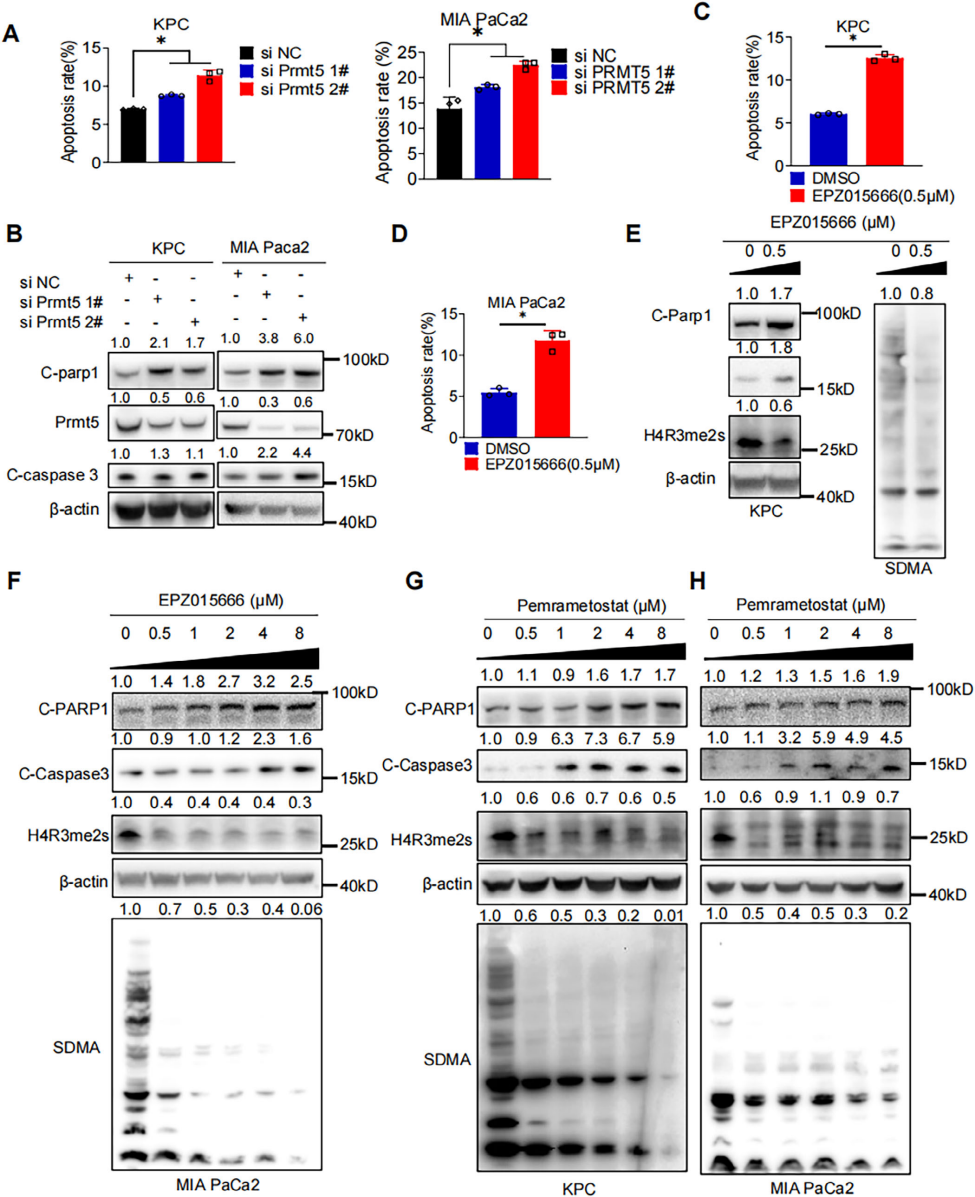
PRMT5 Inhibition Induced Apoptosis of PAAD Cells in Vitro A. The apoptosis statistics of PAAD cells (KPC, left; MIA PaCa2, right) with genetic PRMT5 inhibition. B. Western blot detection of apoptosis markers, C‐Caspase3 and C‐PARP1, in PAAD cells (KPC, left. MIA PaCa2, right) with genetic PRMT5 inhibition. C. The apoptosis statistics of KPC with pharmacological PRMT5 inhibition (EPZ015666, 0.5µM) was measured by flow cytometry (PI and annexin VFITC double staining). D. The apoptosis statistics of MIA PaCa2 with pharmacological PRMT5 inhibition (EPZ015666, 0.5µM) was measured by flow cytometry (PI and annexin VFITC double staining). E. Western blot detection of apoptosis markers C‐Caspase3 and C‐PARP1, PRMT5 inhibition marker H4R3me2s and SDMA in KPC with EPZ015666 treatment. F. Western blot detection of apoptosis markers C‐Caspase3 and C‐PARP1, PRMT5 inhibition marker H4R3me2s and SDMA in MIA PaCa2 with EPZ015666 treatment. G. Western blot detection of apoptosis markers C‐Caspase3 and C‐PARP1, PRMT5 inhibition marker H4R3me2s and SDMA in KPC with Pemrametostat treatment. H. Western blot detection of apoptosis markers C‐Caspase3 and C‐PARP1, PRMT5 inhibition marker H4R3me2s and SDMA in MIA PaCa2 with Pemrametosta treatment. Unless specifically indicated, bars represent mean ± SD of technical replicates, p value measured by unpaired T‐test and * represent values of < 0.05. PAAD, pancreatic ductal adenocarcinoma.

To confirm whether inhibition of PRMT5 could induce apoptosis in PAAD cells, we measured the apoptosis after genetic or pharmacological inhibition of PRMT5 in PAAD cells. As shown in Figures 3A and S3A, increased apoptosis was observed in PAAD cells with genetic inhibition of PRMT5. Increased protein expression of apoptosis marker Cleaved‐PARP1 (C‐PARP1) and Cleaved‐Caspase‐3 (C‐Caspase3) were detected by western blot (Figures 3B and S3B). Consistently, increased apoptosis was detected in PAAD cells after treatment with PRMT5 inhibitors (Figures 3C,D  and S3C,D). Western blot detected the increase of C‐PARP1 and C‐Caspase3 in a dose‐dependent manner, while H4R3me2s and SDMA decreased, markers of PRMT5 activity (Figures 3E‐H and S3E). Collectively, PRMT5 inhibition in both genetic and pharmacological manner induces apoptosis in PAAD cells.

### 2.4 PRMT5 Inhibition Induces DNA Replication Stress in PAAD Cells

**FIGURE 4:**
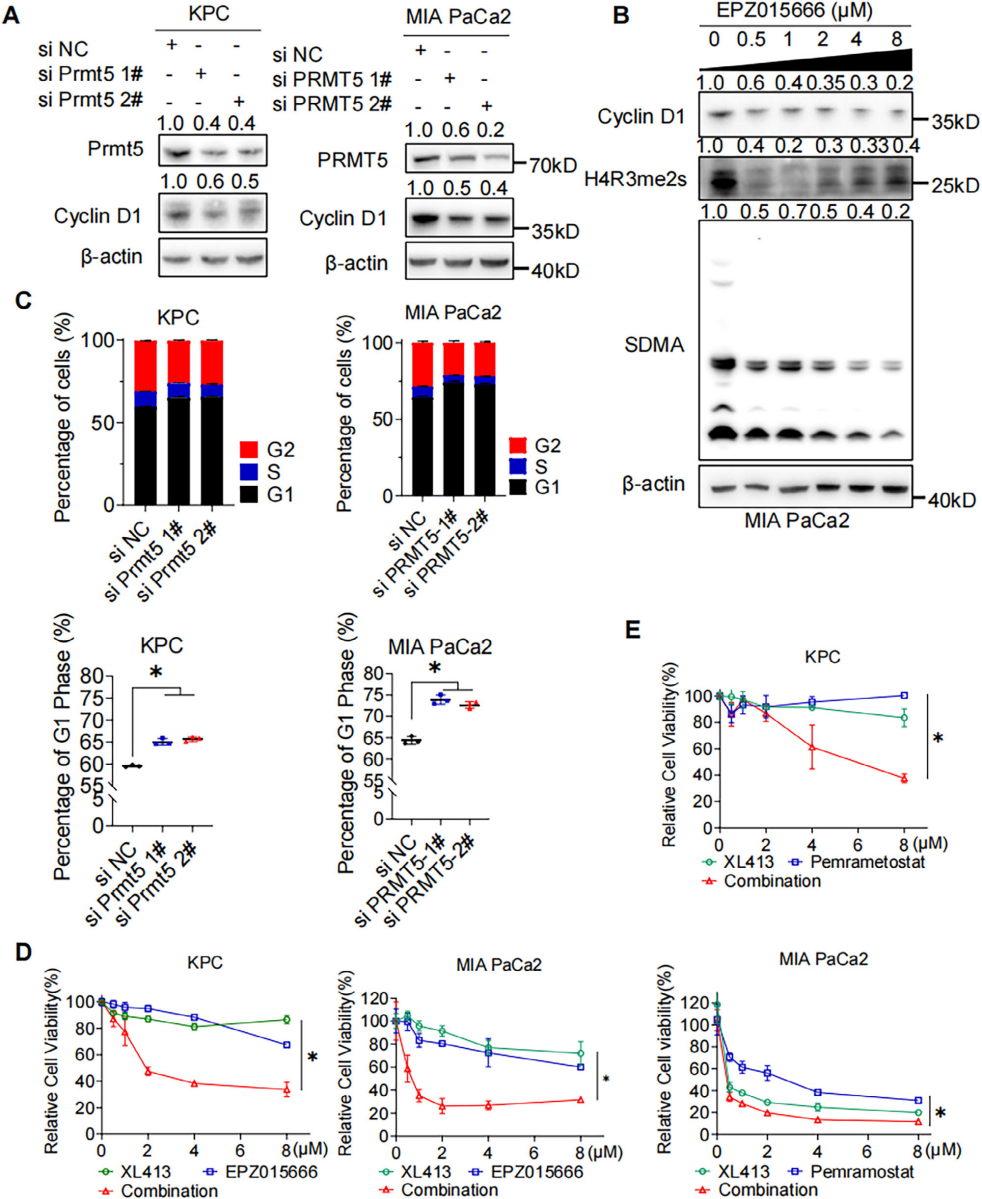
Inhibition of PRMT5 Induces DNA Replication Stress in PAAD Cells. (A) Western blot detection of cell cycle marker cyclin D1 in PAAD cells (KPC, MIA PaCa2) with genetic PRMT5 inhibition. (B) Western blot detection of cyclin D1, H4R3me2s, and SDMA in MIA PaCa2 with pharmacological inhibition of PRMT5 (EPZ015666, 0.5µM to 8µM, 5 days). (C) Flow cytometry detection of cell cycle in PAAD cells (KPC, MIA PaCa2) with genetic PRMT5 inhibition. (D) Cell viability of PAAD cells (KPC, left. MIA PaCa2, right) after administration of EPZ015666, CDC7 inhibitor XL413, and combination was measured by MTS assay. (E) Cell viability of PAAD cells (KPC, upper. MIA PaCa2, lower) after administration of Pemrametostat, CDC7 inhibitor XL413, and combination was measured by MTS assay. Unless specifically indicated, bars represent mean ± SD of technical replicates, p value measured by unpaired T‐test and * represent values of < 0.05. PAAD, pancreatic ductal adenocarcinoma.

To further investigate the function of PRMT5 in PAAD cells, we assessed the cell cycle change after PRMT5 inhibition. Western blot detection exhibited decreased expression of G1 phase marker cyclinD1 after PRMT5 knockdown in PAAD cells (Figures 4A and S4A), indicating DNA replication stress in G1 phase. Similarly, administration of EPZ015666 decreased Cyclin D1 expression in a dose‐dependent way in MIA PaCa2 (Figure 4B). Consistently, flow cytometry assay of cell cycle displayed significantly increased percentage of G1 phase (Figure 4C). XL413 is a potent and selective inhibitor of cell division cycle 7 homolog (CDC7) kinase, blocking DNA replication during the S phase and delaying progression through the G2/M phase of the cell cycle [21, 22]. Excitingly, coadministration of PRMT5 inhibitors and XL413 exhibited synergistic effect to suppress PAAD proliferation (Figure 4D,E). Increased percentage of G1 phase and G2 phase were detected (Figure S4B–D). These results indicates that PRMT5 promotes cell cycle progression, and combination of PRMT5 inhibitors with cyclin‐dependent kinase (CDK) inhibitors may provide therapeutic value in pancreatic cancer.

### 2.5 PRMT5 Activates MYC Hallmark Targets and Increases the Expression of c‐Myc by Transcription Activation

**FIGURE 5:**
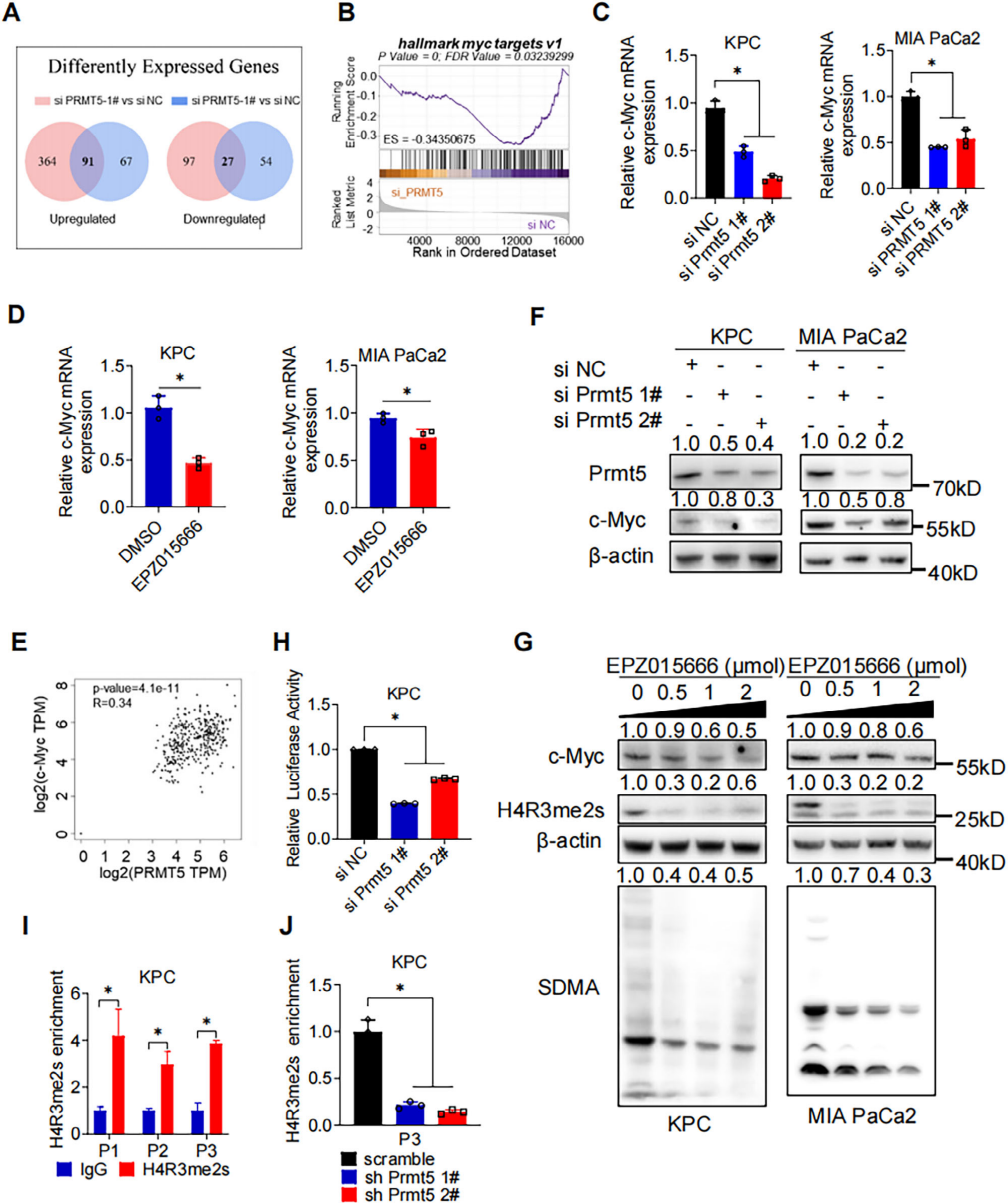
PRMT5 Increases the Expression of c‐Myc by Transcription Activation. (A) Significant differently expressed genes pooling strategy between PRMT5 knockdown and control groups. (B) GESA analysis of differently expressed genes (DEGs). (C) The mRNA level of c‐Myc after genetic PRMT5 inhibition was measured by qRT‐PCR. (D) The mRNA level of c‐Myc after pharmacological PRMT5 inhibition was measured by qRT‐PCR. (E) Correlation between PRMT5 and c‐Myc was analyzed by Pearson method. (F) Western blot detection of c‐Myc in PAAD cells (KPC, left; MIA PaCa2, right) after genetic PRMT5 inhibition. (G) Western blot detection of c‐Myc, H4R3me2s, and SDMA in PAAD cells (KPC, left; MIA PaCa2, right) after pharmacological PRMT5 inhibition. (H) Transcription activity of c‐Myc in KPC after genetic PRMT5 inhibition was measured by dual luciferase reporter assay. (I) The mRNA level of H4R3me2s in the promoter region of c‐Myc was measured by qRT‐PCR. (J) The mRNA level of H4R3me2s in the promoter region of c‐Myc after genetic PRMT5 inhibition in KPC was measured by qRT‐PCR. P1, primer 1. P2, primer 2. P3, primer 3. Unless specifically indicated, bars represent mean ± SD of technical replicates, p value measured by unpaired T‐test and * represent values of < 0.05. PAAD, pancreatic ductal adenocarcinoma.

To determine the target genes and potential regulation mechanism of PRMT5, we performed RNA sequencing to profile different expressed genes before and after PRMT5 knockdown. Bioinformatic analysis revealed 91 genes were upregulated and 27 genes were downregulated after PRMT5 silencing (Figure 5A). Gene Set Enrichment Analysis revealed that hallmark MYC targets were downregulated after PRMT5 depletion (Figure 5B). Both genetic and pharmacological inhibition of PRMT5 downregulated the mRNA level of c‐Myc in PAAD cells. (Figure 5C,D). Further analysis based on TCGA PAAD dataset (GEPIA2 platform) showed the positive correlation between c‐Myc and PRMT5 in mRNA level (Figure 5E).

In order to verify the RNA sequencing results, we performed the qRT‐PCR to assess the expression levels of c‐Myc in PAAD cells after PRMT5 inhibition. Both genetic and pharmacological inhibition of PRMT5 reduced protein expression of c‐Myc (Figure 5F,G). These results indicated that PRMT5 regulated c‐Myc at mRNA level. We designed the c‐Myc plasmids and performed dual luciferase report assay in KPC cells with or without PRMT5 knockdown. The luciferase activity significantly decreased after PRMT5 knockdown, indicating reduced transcription activity of c‐Myc (Figure 5H). We then designed three primers for H4R3me2s in the promoter region of c‐Myc and performed chromatin immunoprecipitation (ChIP) assay accordingly. As shown in Figure 5I,J, H4R3me2s significantly enriched in the promoter region of c‐Myc in KPC and significantly decreased after PRMT5 knockdown. These results indicated that PRMT5 could directly bind to the promoter region of c‐Myc and activate its transcription. Taken together, PRMT5 activates MYC and regulates the expression of c‐Myc by direct binding to the promoter region and subsequent transcription activation, thus promoting PAAD tumorigenesis.

### 2.6 c‐Myc Maintains the Expression of PRMT5 by Enhancing its Protein Stability

**FIGURE 6:**
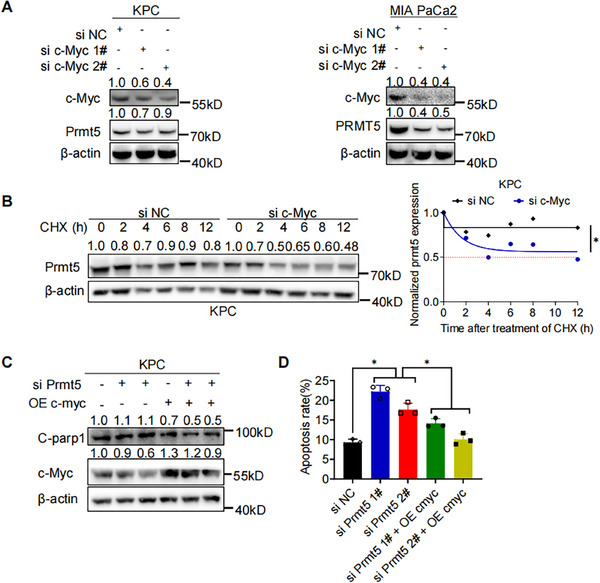
c‐Myc Maintains the Expression of PRMT5 by Enhancing Its Protein Stability. (A) Western blot detection of PRMT5 in PAAD cells (KPC, left. MIA PaCa2, right) c‐Myc knockdown. (B) The half‐life of PRMT5 in KPC with or without c‐Myc knockdown via siRNAs was detected by western blot and the half‐life of PRMT5 in KPC with or without c‐Myc knockdown via siRNAs was determined by ImageJ software. (C) Western blot detection of C‐parp1, c‐Myc in KPC with or without concomitant c‐Myc overexpression and PRMT5 knockdown. (D) Flow cytometry detection of apoptosis in KPC with or without concomitant c‐Myc overexpression and PRMT5 knockdown. Unless specifically indicated, bars represent mean ± SD of technical replicates, p value measured by unpaired T‐test and * represent values of < 0.05. PAAD, pancreatic ductal adenocarcinoma.

Luo et al found that c‐Myc induced PRMT5 expression in liver cancer, we thus questioned whether c‐Myc could regulate the expression of PRMT5 in PAAD [23]. c‐Myc knockdown reduced the protein level of PRMT5 rather than the mRNA level, indicating it might affect the translation or PTM of PRMT5 (Figures 6A and S6A). We then administered the protein translation inhibitor CHXT to KPC with or without c‐Myc knockdown. Western blot detection revealed shortened half‐life of PRMT5 in KPC with c‐Myc knockdown (Figure 6B), indicating enhanced protein degradation. Therefore, we performed protein degradation assay. After administration of proteasome inhibitor MG132, increased PRMT5 was detected in KPC with c‐Myc knockdown (Figure S6B). we further asked whether c‐Myc overexpression could rescue the apoptosis resulted from PRMT5 knockdown. Intriguingly, after c‐Myc overexpression, less expression of C‐PARP1 and apoptosis was detected in KPC with PRMT5 knockdown (Figure 6C,D). Taken together, these results strongly supported that PRMT5 formed a positive feedback loop with c‐Myc to exert its proliferation‐promoting role in PAAD.

### 2.7 Inhibition of PRMT5 Impedes PAAD Development in Vivo

**FIGURE 7:**
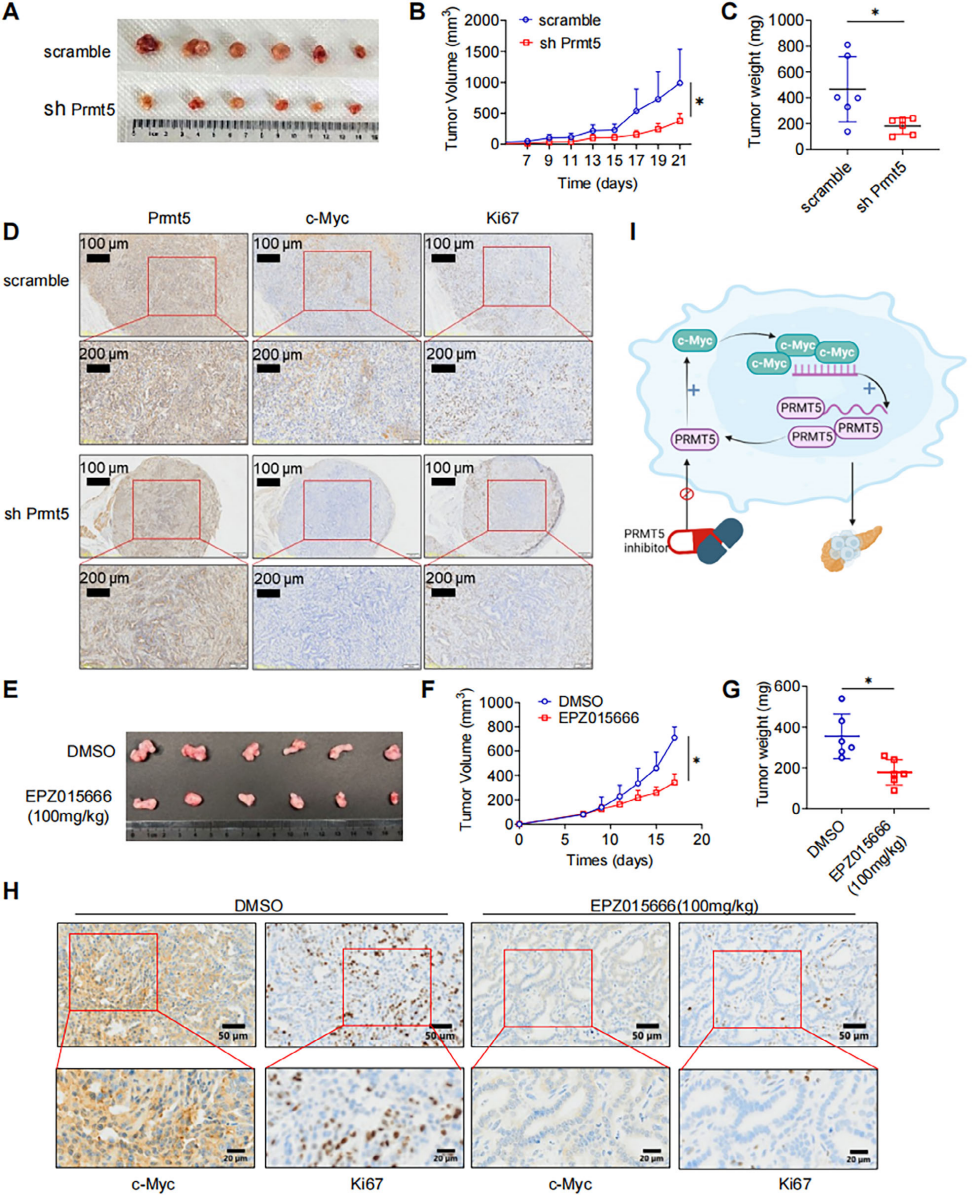
Inhibition of PRMT5 Impedes PAAD Development in vivo. (A) Representative images displaying tumors harvested from mice bearing sh‐scramble‐ or sh‐Prmt5‐ KPC cells. (B) Tumor volumes were measured at specified time points and dissected at the endpoint. (C) Tumor weights were measured at endpoint. (D) Typical IHC staining for PRMT5, c‐Myc, and Ki67 in tumors of scramble group and sh‐PRMT5 group. (E) Representative images displaying tumors harvested from mice bearing KPC cells treated with DMSO or PRMT5 inhibitors (n=6). (F) Tumor volumes were measured at specified time points and dissected at the endpoint. (G) Tumor weights were measured at endpoint. (H) Typical IHC staining for c‐Myc and Ki67 in tumors of DMSO group and PRMT5 inhibitor group. (I) Working model. PRMT5 increases the expression of oncogene c‐Myc, and c‐Myc increases the expression of PRMT5, forming a positive feedback loop and promoting the tumorigenesis of pancreatic cancer. Targeting this positive feedback loop with PRMT5 inhibitors can induce cell cycle arrest, apoptosis and suppress proliferation, providing therapeutic effect. Unless specifically indicated, bars represent mean ± SD of technical replicates, p value measured by unpaired T‐test and * represent values of < 0.05. PAAD, pancreatic ductal adenocarcinoma.

To further evaluate the role of PRMT5 in PAAD in vivo tumorigenesis, we knocked down the PRMT5 in KPC cell line with shRNA and established the xenograft model (Figure S7A–C). Consistent with in vitro results, in vivo tumor growth was significantly impaired in PRMT5 depletion group comparing with that in the scramble group (Figure 7A–C). IHC staining showed decreased expression of PRMT5, c‐Myc, and Ki67 in PRMT5 depletion group (Figures 7D and S7D).

Next, we evaluated the therapeutic efficacy of PRMT5 inhibitor in PAAD tumorigenesis. As shown in Figure S7E, xenograft model bearing KPC cells were treated with dimethylsulfoxide (DMSO) or EPZ015666 (100 mg/kg, TIW, six times in total) by regular oral gavage. Consistent with the results of PRMT5 depletion, PRMT5 inhibition significantly impeded tumor growth (Figure 7E–G). Consistently, the expression of c‐Myc and Ki67 were decreased in EPZ015666 treatment (Figures 7H and S7F). Collectively, both genetic and pharmacological inhibition of PRMT5 impedes PAAD tumorigenesis and can be a potential treatment choice for PAAD with high PRMT5 expression.

## 3 Discussion

Protein arginine methylation, catalyzed by PRMTs, has been reported to play a crucial role in tumorigenesis and tumor development [24]. However, the roles and underlying mechanisms of PRMTs in pancreatic cancer have not been fully studied. In this study, we revealed the protumor role of type II PRMTs PRMT5 and its close correlation with poor prognosis in PAAD patients. Mechanistically, PRMT5 could bind to the promoter region of c‐Myc directly and activate its transcription. In turn, increased c‐Myc inhibited proteasome‐mediated degradation of PRMT5, maintaining its expression in pancreatic cancer. By forming this positive PRMT5/c‐Myc feedback loop, PRMT5 fueled the proliferation of pancreatic cancer (Figure 7I).

Type II methyltransferase PRMT5 has been reported to affect cancer biological functions in multiple manners, like histone methylation, inhibition of tumor suppressors or promotion of oncogenes, thus driving cancer cell growth, metastasis, and treatment resistance [23, 25, 26, 27]. For example, PRMT5 mediated the methylation of the arginine residue of E2F1 [28]. PRMT5 catalyzed symmetric demethylation of AKT1 at arginine 391 and promoted the activation of AKT to exert oncogenic function [29]. In leukemia and lymphoma cells, by methylation of the promoter region, PRMT5 induced silence of tumor suppressors Rb and enhanced proliferation [30]. In lung cancer, PRMT5 suppressed the transcription of miR‐99 by SDMA of histone H4R3, leading to increased expression of epidermal growth factor receptor 3 and activation of Erk1/2 and Akt, thus facilitating the metastasis [31].

Accumulating evidence suggested that PRMT5 functioned as an oncogenic role in pancreatic cancer. PRMT5‐mediated methylation suppressed the autophosphorylation and kinase activity of Hippo pathway initiator MST2, resulting in the inactivation of the tumor suppressor pathway Hippo signaling axis and progression in pancreatic cancer [12]. By promoting the EMT, PRMT5 enhanced metastasis capability of pancreatic cancer. Combination of PRMT5 inhibition exhibited synergistically enhanced cytotoxicity of gemcitabine and palbociclib in pancreatic cancer [14, 15]. PRMT5 was identified as an indirect target of CDK4 and was essential for CDK4/6 inhibitor sensitivity. In the present study, we investigated the effect of PRMT5 on cell cycle in pancreatic cancer. Interestingly, PRMT5 inhibition induced DNA replication stress in pancreatic cancer, and coadministration of PRMT5 inhibitor with CDC7 inhibitor XL413 exhibited promising therapy value.

Consistent with reported studies [32], PRMT5 knockdown or inhibition induced anti‐PAAD subtypes including suppressed colony formation, suppressed proliferation, increased apoptosis process, and prolonged survival of tumor‐bearing mouse. We further performed RNA‐sequencing on PRMT5‐knockdown pancreatic cancer cells, inhibited in vivo tumor growth, and revealed the positive regulation of cell death, positive apoptosis process, and enriched Hallmark of apoptosis after PRMT5 knockdown. These results were consistent with its clinicopathological features.

The regulation between PRMT5 and c‐Myc has been reported by several publications; however. the focuses and the exact molecular mechanisms are much different. Liang et al. [18] reported that PRMT5 activated lipid metabolic reprogramming through MYC, contributing to the growth and survival of mantle cell lymphoma, while the exact regulation mechanism between PRMT5 and MYC is less addressed. Similarly, although Zhang et al. [33] claimed that PRMT5 activated c‐Myc through up‐regulating NF‐kappaB pathway, the detailed mechanism research was lacking. Liu et al. [17] reported that PRMT5 directly bound c‐Myc, and this binding was required to the transcriptional repression of c‐Myc target genes in gastric cancer. Qin et al. [16] proposed the PRMT5/FBW7/c‐Myc axis in their work. They found that PRMT5 posttranslationally regulated c‐Myc stability via the E3 ubiquitin ligase FBW7, which controlled c‐Myc degradation, thus increasing c‐Myc levels and leading to the subsequent enhanced proliferation and aerobic glycolysis in pancreatic cancer cells. In this study, we found that PRMT5 regulated the expression of c‐Myc at mRNA level. By direct binding to the promoter region of c‐Myc, PRMT5 activated its transcription and increased its expression. The increased c‐Myc inhibited the proteasome‐mediated degradation of PRMT5, maintaining its expression in pancreatic cancer. Intriguingly, overexpression of c‐Myc rescued the apoptosis induced by PRMT5 knockdown. Taken together, by forming this positive feedback loop, PRMT5 functioned its oncogenic role and fueled the proliferation of pancreatic cancer.

There are several limitations in this study. First, the cohort size is limited. Since most pancreatic cancer patients are diagnosed at the advanced stage, it is only possible to get enough surgical specimens in limited medical centers in China, like West China Hospital of Sichuan University and Fudan University Shanghai Cancer Center. Thus, we applied commercial tissue array to detect the expression level of PRMT5 in pancreatic cancer and adjacent normal pancreas. Second, to better understand the intrinsic versus extrinsic functions of PRMT5, transgenic mice model or PRMT5 knock‐out model by CRISPR Cas9 technology are recommended. Third, to get more solid evidence and generalize our findings in different clinical settings, clinical trials are recommended. Although these are beyond our current capabilities, we are deeply interested and determined to carry out related work step by step.

## 4 Conclusions

In summary, we postulated and validated the novel positive PRMT5/c‐Myc feedback loop via which PRMT5 exerted its pro‐proliferation role in pancreatic cancer. Targeting this communication may be a potential therapy choice. In addition, our study supported with previous publication that PRMT5 could be a novel marker for pancreatic cancer patients’ classification and potential therapeutic target, providing preclinical evidence that PRMT5 could be translated into a therapeutic target for treatment for pancreatic cancer.

## 5 Materials and Methods

### 5.1 siRNAs shRNA and Small Molecule Inhibitors

siRNA targeting PRMT5, c‐Myc were synthesized by GenePharma (Shanghai, China). SiRNAs were transfected by Lipofectamine RNAiMAX transfection reagent (Invitrogen, USA) according to the manufacturer's protocol. ShRNA were synthesized by OBiO (Shanghai, China). The sequence of used siRNAs and shRNA was listed in Table S2. Polybrene (Sigma–Aldrich; TR‐1003‐G, final concentration 5–10 µg/mL) were used to increase the efficiency of viral infection. Reagents used in this study were as follows: Pemramostat (S8664; Selleck, USA), EPZ015666 (S7748; Selleck), XL413 (catalog no. HY‐15260; MCE, USA), cycloheximide (S7418, USA), and MG132 (Calbiochem, USA; 474790).

### 5.2 Cell Culture

MIA PaCa2, BxPC‐3, PANC‐1, and AsPC‐1 were obtained from the Type Culture Collection of the Chinese Academy of Sciences (Shanghai, China). Murine pancreatic cancer cell KPC and normal pancreatic duct cell hTERT–HPNE were kind gifts from Dr Yi Wang. BxPC‐3 was cultured in RPMI medium 1640 (Cat.6124133) containing 10% (vol/vol) fetal bovine serum (FBS) and 1% penicillin/streptomycin. Other cell lines were cultured in Dulbecco's modified Eagle medium (Cat. 6124167; Gibco) containing 10% (vol/vol) FBS and 1% penicillin/streptomycin. Cells were cultured in a humidified incubator at 37°C and 5% CO_2_. Cell lines were tested for mycoplasma contamination regularly by PCR and discarded if positive.

### 5.3 RNA Isolation and PCR

Trizol reagent (Invitrogen) and NanoDrop 2000 (Nanodrop, USA) were used to extract and quantify the total RNA. 1 µg total RNA was reverse transcribed with Hifair® AdvanceFast One‐step RT‐gDNA Digestion SuperMix for qPCR (Cat. 11151ES60; Yeasen Biotechnology, Shanghai, China). Quantitative real‐time PCR was performed to determine the relative expression of mRNA level with the Hieff UNICON® Universal Blue qPCR SYBR Green Master Mix (Cat. 11184ES08; Yeasen Biotechnology). The primers were displayed in Table S3.

### 5.4 Immunohistochemistry

IHC were carried out as previously reported [34]. Primary antibodies were listed in Table S4. IHC staining pictures in Figure S1F were downloaded from The Human Protein Atlas [20]. Protein expression levels were determined by assessing the intensity of staining (0, pale; 1, mild; 2, moderate; 3, intense), and the percentage of positive cells of the total cells in each field (0, <1%; 1, 1–25%; 2, 26–50%; 3, 51–75%; 4, 76–100%). Multiplied score was used to represent the protein expression, which was calculated as multiplied score = intensity of staining × percentage × 100%, high or low status were defined based on the comparison with the median value.

### 5.5 Immunoblotting

RIPA buffer supplemented with protease inhibitors were used to extract the total proteins, which were quantified with BSA standard methodology as previously reported [35]. Primary antibodies against PRMT5 (18436‐1‐AP; Proteintech), c‐Myc (67447‐1‐Ig; Proteintech), H4R3me2s (A‐3718‐050; EPIGENTEK), Cyclin D1 (2978; Cell Signaling Technology), C‐PARP (9544; Cell Signaling Technology), C‐Caspase3 (9664; Cell Signaling Technology), and β‐actin (4967; Cell Signaling Technology) were used for western blots. All western blots were repeated at three times.

### 5.6 Cell Viability Assay

PAAD cells were plated at a density of 1 × 10^3^ cells per well into 96‐well plates. Cells were treated with PRMT5 inhibitors (Pemramostat or EPZ015666; 0.5, 1, 2, 4, and 8 µM), or transfected with PRMT5 siRNAs/shRNAs. Cell viability was assessed using the MTS Kit (Cat. ST1009; Saint‐bio, Shanghai, China) at 0, 24, 48, 72, 96, and 120 h, following the manufacturer's protocol.

### 5.7 Apoptosis and Cell Cycle Assay

Cell apoptosis was evaluated using flow cytometry and western blotting. For flow cytometry, cells were collected and resuspended in 100 µL of 1× binding buffer. Then, 5 µL of fluorescein isothiocyanate (FITC) annexin V and propidium iodide (PI) (Cat. AT101‐AT1; Multisciences (Lianke) Biotech Co., Ltd, China) were added to the suspension and incubated for 15 min at room temperature. After incubation, 400 µL of 1× binding buffer was added, and samples were analyzed using a Cytek Aurora CS flow cytometer (Aurora CS; S0165). The cell cycle was analyzed using flow cytometry with the Cell Cycle Staining Kit (Cat. CCS012; Multisciences (Lianke) Biotech Co., Ltd, China), following the provided protocol.

### 5.8 Luciferase Reporter Assay

The plasmid of c‐Myc was generated by Tsingke Biotech Co., Ltd. The plasmid was cotransfected with pRL renilla and prmt5 siRNA using Lipo3000 (Invitrogen) or treated with pyrvinium using X‐treme GENE HP DNA Transfection Reagent (Roche, USA). Forty‐eight hours later, luciferase activity in the cell lysates was measured using the Dual‐Glo Luciferase Assay System (E2920; Promega) following the manufacturer's protocol.

### 5.9 Chromatin Immunoprecipitation

The ChIP assay was performed using the SimpleChIP Enzymatic Chromatin IP Kit (CST, USA; 91820S) with anti‐H4R3me2s antibody (Abcam; AB5823). The primers used for the qPCR analysis of precipitated DNA were listed in Supporting Information.

### 5.10 Animal Experiments

All animal experiments were approved by the Institutional Animal Care and Use Committee, Zhejiang Center of Laboratory Animals (ZJCLA) (ZJCLA‐IACUC‐20020055). Six‐week‐old male BALB/c athymic nude mice (nu/nu, *n* = 6 per group) were purchased from the ZJCLA and used with each experimental group. For in vivo genetic inhibition of PRMT5, 1 × 10^6^ KPC scramble/shPrmt5 cells was resuspended in a total volume of 100 µL 1× PBS and injected subcutaneously into the upper right flank of mice. For pharmacological inhibition of PRMT5 in vivo, 1 × 10^6^ KPC cells was resuspended in a total volume of 100 µL 1× PBS and injected subcutaneously in the same way. Tumor sizes were regularly monitored using calipers. Three weeks after injection, mice were euthanized with CO_2_, tumors were harvested, weighed, and assessed for further experiments. The PRMT5 inhibitors Pemramostat/EPZ015666 were prepared in DMSO and corn oil, and administered to mice (100 mg/kg, oral gavage, TIW, six times in total).

### 5.11 TCGA Data Analysis

Using the gene expression profiling interactive analysis (GEPIA2, http://gepia2.cancer‐pku.cn) [36], we integrated the differential gene expression and patient survival data from the TCGA cohort. The correlation between PRMT5 mRNA and PAAD grade was analyzed on TISIDB database [19]. OS and DFS were calculated based on the Kaplan–Meier method with a 50% median cutoff and compared using the log‐rank test.

### 5.12 Statistical Analysis

Statistical analysis was conducted using Prism software (version 8.0.2, GraphPad) and SPSS 22.0. Data are presented as mean ± SD, with the exact sample size (*n*) for each experiment indicated in the figure legend. All measurements were repeated at least three times, showing consistent trends. Unless stated otherwise, statistical significance was analyzed using the Student's *t*‐test, with a *p* value < 0.05 considered significant (noted with *). Outlier data, defined as values deviating from the mean by more than two standard deviations, were excluded from the analysis.

## Author Contributions

*Conceptualization*: Y. F., S. P., X. X., and W. K. *Methodology*: Y. F., S. P., X. Z. F., and S. R. Y. *Investigation*: Y. F., S. P., X. Z. F., and S. R. Y. *Writing—original draft*. Y. F. and S. P. *Writing—review and editing*: Y. F., S. P., X. F., W. Y. C., X. X., and W. K. *Supervision*: X. X. and W. K. *Funding acquisition*: Y. F., S. P., and X. X.

## Conflicts of Interest

The authors declare no conflicts of interest.

## Ethics Statement

Tissue arrays (HPanA180Su03) were obtained from Shanghai Outdo Biotech Company. Research involving human tissues was approved by the Human Ethics Review Committee of Shanghai Outdo Biotech Company (SHYJS‐CP‐1901009/YB M‐05‐02), in accordance with the Declaration of Helsinki guidelines. The study also complies with all relevant ethical regulations for human research as set forth by the Human Ethics Review Committee of the Affiliated Hangzhou First People's Hospital, School of Medicine, Westlake University. Written informed consent was waived for the retrospective use of patients’ surgical specimens (KY‐2024302‐01). All animal experiments were conducted with approval of the Institutional Animal Care and Use Committee, ZJCLA (ZJCLA‐IACUC‐20020055).

## Consent

The authors have nothing to report.

## Supporting information



Supporting Information

## Data Availability

Bulk‐RNAseq data generated are uploaded to NCBI‐GEO, and other datasets used and/or analyzed are available upon reasonable request to the corresponding author.
