# Supplementary material for: PRMT5 Promotes Pancreatic Cancer Tumorigenesis via Positive PRMT5/C‐Myc Feedback Loop
Source: MedComm (2020). 2025 May 15;6(6):e70150. doi: 10.1002/mco2.70150 (PMC12079021; doi:10.1002/mco2.70150)
Supplement: Supplementary file 1 — Supporting Information [file MCO2-6-e70150-s001.docx]

PRMT5 Promotes Pancreatic Cancer Tumorigenesis via Positive PRMT5/C-Myc Feedback Loop

Fan Yang^1, #^, Ping Song^2,3,4, #^, Zhaofeng Xiao^5^, Renyi Su^9^, Xin Fang^1^, Yichao Wu^6^, Xiao Xu^6,7,8, *^, Kai Wang^8*^

^1^Department of Vascular Surgery, Affiliated Hangzhou First People’s Hospital, School of Medicine, Westlake University, Hangzhou, 310006, China.

^2^Department of Gastroenterology, Affiliated Hangzhou First People’s Hospital, School of Medicine, Westlake University, Hangzhou, 310006, China.

^3^Key Laboratory of Integrated Traditional Chinese and Western Medicine for Biliary and Pancreatic Diseases of Zhejiang Province, Hangzhou, 310006, China.

^4^Hangzhou Institute of Digestive Diseases, Hangzhou, 310006, China.

^5^The Fourth School of Clinical Medicine, Zhejiang Chinese Medical University, Hangzhou, 310053, China.

^6^Department of Hepatobiliary, Pancreatic and Minimal Invasive Surgery, Zhejiang Provincial People’s Hospital (Affiliated People’s Hospital), Hangzhou Medical College, Hangzhou, 310014, China.

^7^NHC Key Laboratory of Combined Multi-Organ Transplantation, Hangzhou, 310003, China.

^8^School of Clinical Medicine, Hangzhou Medical College, Hangzhou, 310059, China.

^9^School of Medicine, Zhejiang University, Hangzhou, 310058, China.

# Fan Yang and Ping Song contributed equally to this study

*** Corresponding author and lead contact:**

Prof. Kai Wang, E-mail: kaiw3@zju.edu.cn

^1^ School of Clinical Medicine, Hangzhou Medical College, Hangzhou, 310059, China.

Prof. Xiao Xu, E-mail: zjxu@zju.edu.cn

^1^Department of Hepatobiliary, Pancreatic and Minimal Invasive Surgery, Zhejiang Provincial People’s Hospital (Affiliated People’s Hospital), Hangzhou Medical College, Hangzhou, 310014, China.

^2^NHC Key Laboratory of Combined Multi-Organ Transplantation, Hangzhou, 310003, China.

^3^School of Clinical Medicine, Hangzhou Medical College, Hangzhou, 310059, China.

**Supplementary materials**

**Supplementary Figure and Figure legend**

**
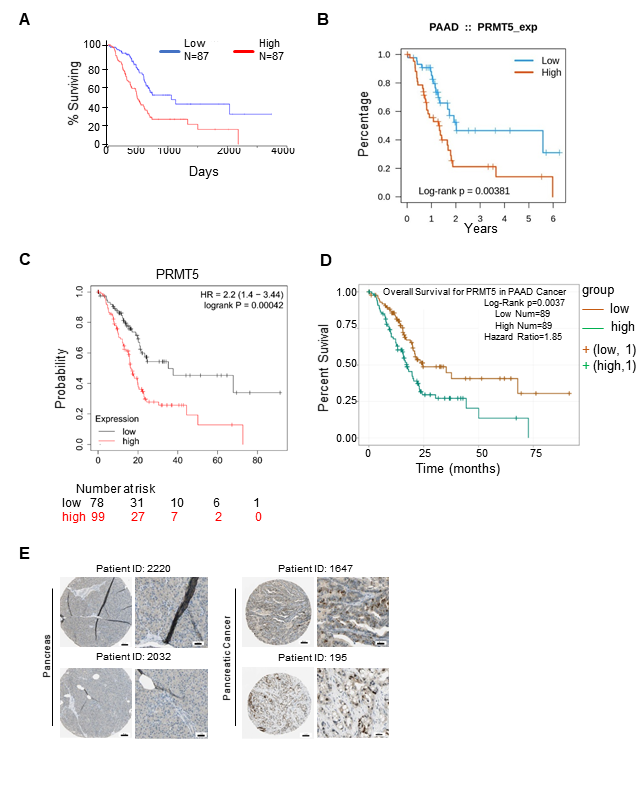
**

**Figure S1. High Expression of PRMT5 Correlates with the Poor Prognosis in** [**Pancreatic Cancer**](javascript:;)

**A-D.** Kaplan-Meier survival analysis of patients’ overall survival (OS) with different PRMT5 mRNA expression. (**A.** COX database; **B.** TISIBD database; **C.** TCGA-PORTAL database; **D.** STARBASE database) **E.** Representative IHC staining images of PRMT5 protein in normal pancreas and pancreatic cancer. (The Human Protein Atlas database).

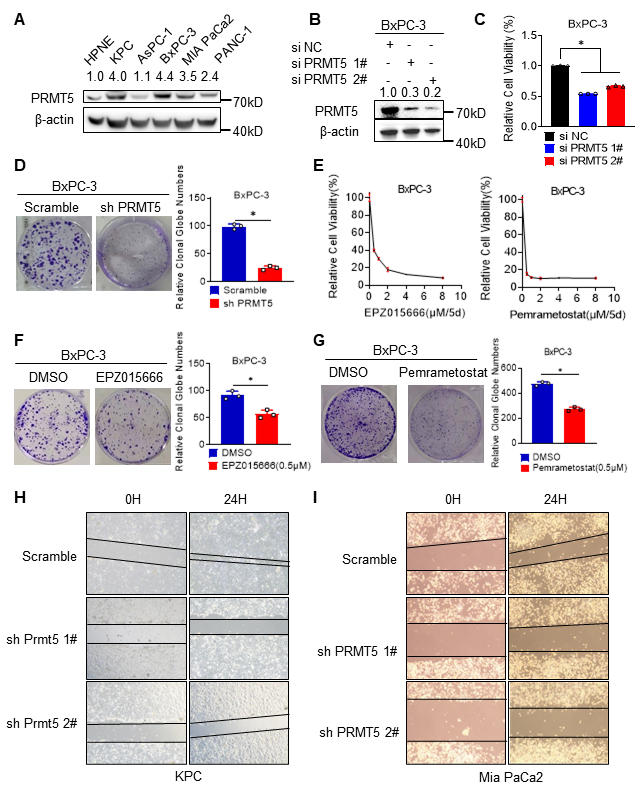


**Figure S2. PRMT5 Promotes Proliferation of Pancreatic Cancer Cells.**

**A.** Western blot detection of PRMT5 in normal pancreatic duct cell and pancreatic cancer cells. From left to right: normal pancreatic duct cell, HPNE. murine pancreatic cancer cell line, KPC. human pancreatic cancer cell lines, AsPC-1, BxPC-3, MIA PaCa2, PANC-1. **B.** Western blot detection of PRMT5 in BxPC-3 with or without PRMT5 knockdown. **C.** Cell viability of BxPC-3 with or without PRMT5 knockdown was measured by MTS assay. **D.** Colony formation assay of BxPC-3 with or without PRMT5 knockdown. **E.** Cell viability of BxPC-3 with or without pharmacological PRMT5 inhibition (EPZ015666, left. Pemrametostat, right) was measured by MTS assay. **F.** Colony formation assay of BxPC-3 with or without EPZ015666 treatment (0.5μM, 14 days) **G.** Colony formation assay of BxPC-3 with or without Pemrametostat treatment (0.5μM, 14 days). **H.** Scratch assay of KPC with or without PRMT5 knockdown. **I.** Scratch assay of MIA PaCa2 with or without PRMT5 knockdown. Unless specifically indicated, bars represent mean ± SD of technical replicates, p value measured by unpaired T-test and * represent values of < 0.05. PAAD, pancreatic ductal adenocarcinoma.


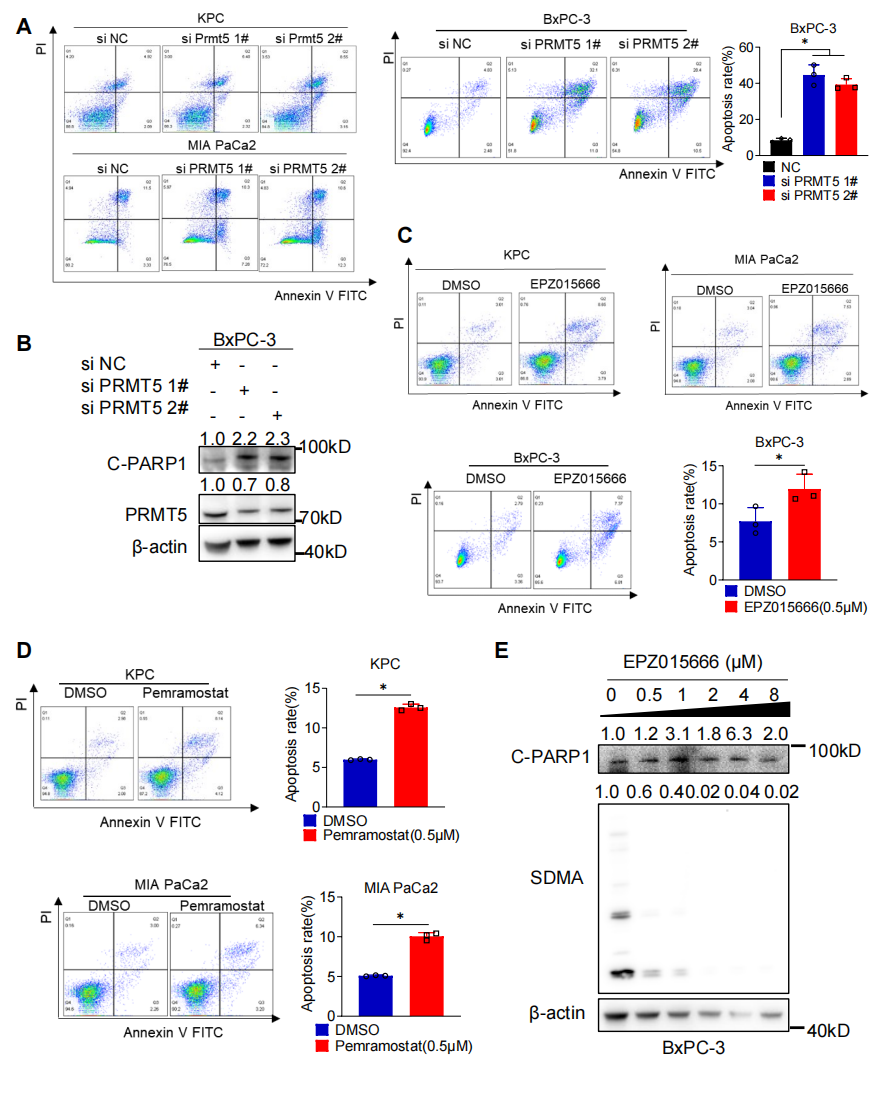


**Figure S3. PRMT5 Inhibition Promotes Apoptosis in PAAD Cells**

**A.** Flow cytometry assay of apoptosis in pancreatic cancer cells (KPC, MIA PaCa2, and BxPC-3) with or without PRMT5 knockdown. **B.** Western blot detection of apoptosis marker C-PARP1 in BxPC-3 with or without PRMT5 knockdown. **C.** Flow cytometry assay of apoptosis in pancreatic cancer cells (KPC, MIA PaCa2, and BxPC-3) with or without EPZ015666 treatment (0.5μM). **D.** Flow cytometry assay of apoptosis in PAAD cells (KPC, upper lane. MIA PaCa2, lower lane). **E.** Western blot detection of C-PARP1 and SDMA in BxPC-3 with or without pharmacological PRMT5 inhibition (EPZ015666, 0.5μM to 8μM, 5 days). Unless specifically indicated, bars represent mean ± SD of technical replicates, p value measured by unpaired T-test and * represent values of < 0.05. PAAD, pancreatic ductal adenocarcinoma.

**
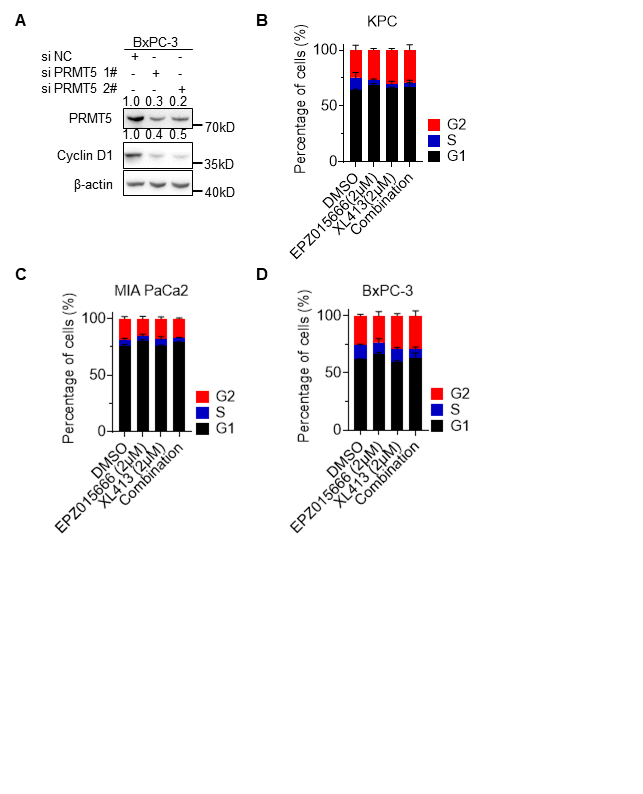
**

**Figure S4. PRMT5 Inhibition Induces Cell Cycle Arrest.**

**A.** Western blot detection of PRMT5 and Cyclin D1 in BxPC-3 with or without PRMT5 knockdown. **B.** Flow cytometry assay of cell cycle in KPC after administration of DMSO, EPZ015666 (2μM), CDC7 inhibitor XL413 (2μM) and combination. **C.** Flow cytometry assay of cell cycle in MIA PaCa2 after administration of DMSO, EPZ015666 (2μM), CDC7 inhibitor XL413 (2μM) and combination. **D.** Flow cytometry assay of cell cycle in BxPC-3 after administration of DMSO, EPZ015666 (2μM), CDC7 inhibitor XL413 (2μM) and combination. Unless specifically indicated, bars represent mean ± SD of technical replicates, p value measured by unpaired T-test and * represent values of < 0.05. PAAD, pancreatic ductal adenocarcinoma.


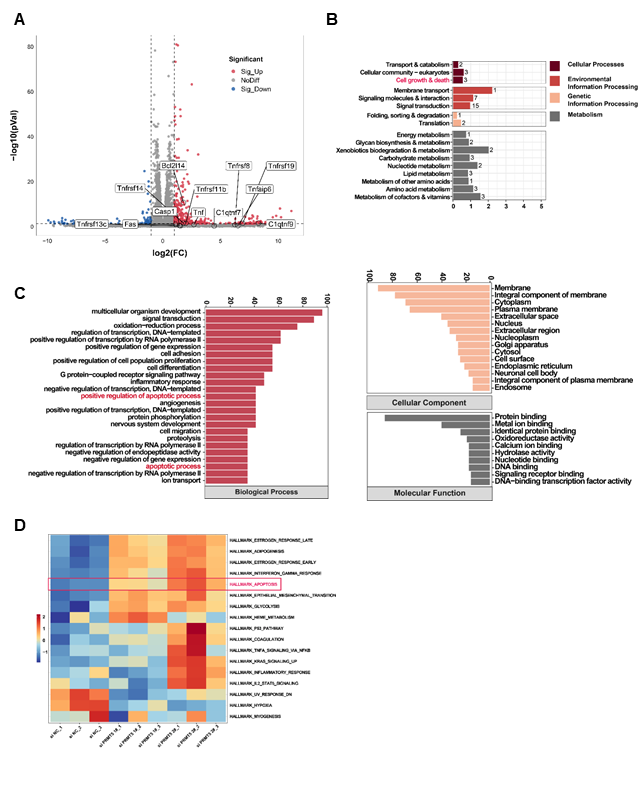


**Figure S5. Bioinformatic Analysis of KPC with or without Genetic PRMT5 inhibition.**

**A.** Volcano plot of differently expressed genes (DEGs). **B.** Kyoto Encyclopedia of Genes and Genomes (KEGG) enrichment analysis of DEGs. **C.** Gene Oncology (GO) enrichment analysis of DEGs. **D.** Gene set variation analysis (GSVA) of DEGs.


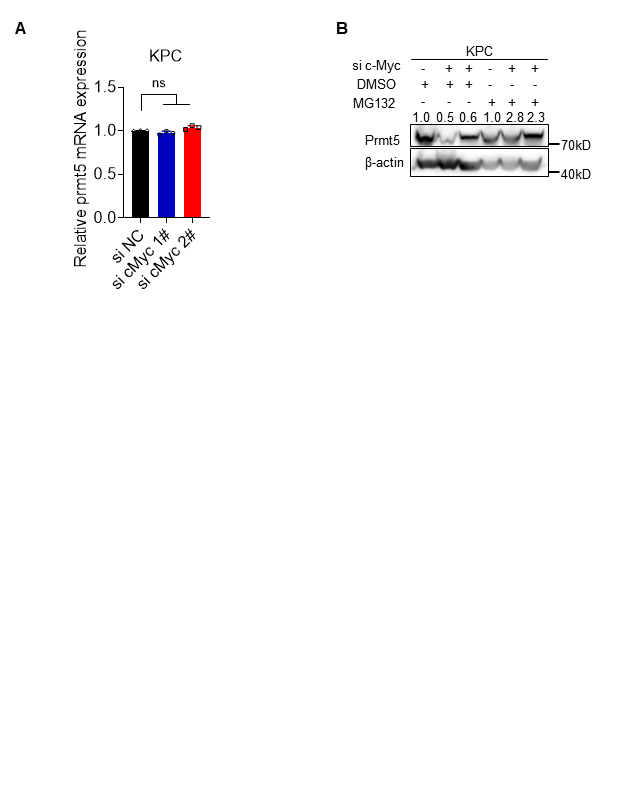


**Figure S6. C-Myc Increases PRMT5 Expression by Inhibition of Proteasome-mediated Degradation.**

**A.** The mRNA level of PRMT5 in KPC with or without c-Myc knockdown was measured by RT-qPCR. **B.** Western blot detection of PRMT5 in KPC with or without c-Myc knockdown. Western blot detection of the expression of PRMT5 in KPC with or without c-Myc knockdown via siRNAs was detected. Unless specifically indicated, bars represent mean ± SD of technical replicates, p value measured by unpaired T-test and * represent values of < 0.05. PAAD, pancreatic ductal adenocarcinoma.


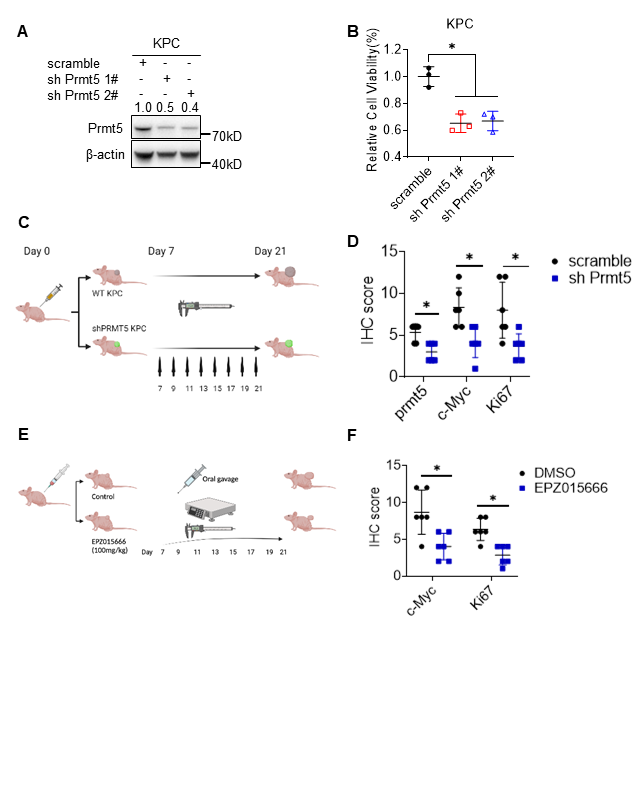


**Figure S7. PRMT5 Inhibition Decreased Expression of c-Myc and Ki-67.**

**A.** Stable PRMT5 knockdown cell line establishment with shRNA. **B.** The cell viability was detected between scramble and shPRMT5 in vitro by MTS. **C.** Schematic protocol of the PRMT5 knockdown sub-cutaneous model and tumor growth curve in immunodeficient mice. (n=6). **D.**IHC score analysis of PRMT5, c-Myc and Ki 67 in tumor samples derived from KPC with or without PRMT5 knockdown. **E.** Schematic protocol of PRMT5 inhibitor sub-cutaneous model and tumor growth curve in immunodeficient mice. **F.** IHC score analysis of c-Myc and Ki 67 in tumor samples with or without pharmacological PRMT5 inhibition. Unless specifically indicated, bars represent mean ± SD of technical replicates, p value measured by unpaired T-test and * represent values of < 0.05. PAAD, pancreatic ductal adenocarcinoma.

**Supplementary table**

| **Supplementary table 1. Clinical characteristics of PAAD tissue array** | | | | | | |
| --- | --- | --- | --- | --- | --- | --- |
| Variables | | PRMT5 expression | | Total  (n=87) | χ2 | p value |
|  |  | low | high |  |  |  |
| Age (year) |  |  |  |  | 0.225 | 0.635 |
|  | ＜60 | 10 | 34 |  |  |  |
|  | ≥60 | 8 | 35 |  |  |  |
| Gender |  |  |  |  | 3.061 | 0.080 |
|  | Female | 4 | 31 |  |  |  |
|  | male | 14 | 38 |  |  |  |
| Grade |  |  |  |  | 0.788 | 0.674 |
|  | I | 8 | 28 |  |  |  |
|  | II | 9 | 32 |  |  |  |
|  | III | 1 | 9 |  |  |  |
| TNM stage |  |  |  |  |  |  |
|  | I | 9 | 15 |  |  | ***0.007 **** |
|  | II | 6 | 21 |  |  |  |
|  | III | 1 | 8 |  |  |  |
|  | IV | 2 | 25 |  |  |  |
| T stage |  |  |  |  |  | 0.205 |
|  | I | 1 | 6 |  |  |  |
|  | II | 10 | 26 |  |  |  |
|  | III | 7 | 26 |  |  |  |
|  | IV | 0 | 11 |  |  |  |
| N stage |  |  |  |  | 7.186 | ***0.028 **** |
|  | N0 | 11 | 21 |  |  |  |
|  | N1 | 4 | 39 |  |  |  |
|  | N2 | 3 | 9 |  |  |  |
| M stage |  |  |  |  | 5.088 | ***0.033 **** |
|  | M0 | 17 | 47 |  |  |  |
|  | M1 | 1 | 22 |  |  |  |
| Vascular invasion |  |  |  |  | 4.247 | ***0.039 **** |
|  | (-) | 14 | 35 |  |  |  |
|  | (+) | 4 | 34 |  |  |  |
| Perineural invasion |  |  |  |  | 1.563 | 0.211 |
|  | (-) | 8 | 20 |  |  |  |
|  | (+) | 10 | 49 |  |  |  |
| lnCEA |  |  |  |  |  | 0.333^#1^ |
|  |  | 1.171±0.767 | 1.461±1.155 |  |  |  |
| lnCA125 |  |  |  |  |  | 0.497^#2^ |
|  |  | 3.451±1.294 | 3.231±0.974 |  |  |  |
| lnCA19-9 |  |  |  |  |  | 0.422^#3^ |
|  |  | 4.757±1.611 | 5.144±1.796 |  |  |  |
| Ki67 |  |  |  |  |  | 0.133^#4^ |
|  | I | 4 | 7 |  |  |  |
|  | II | 4 | 15 |  |  |  |
|  | III | 3 | 13 |  |  |  |
|  | IV | 0 | 5 |  |  |  |
| Note:  *: statistically significant.  #1: 6 patients without CEA data.  #2: 21 patients without CA125 data.  #3: 3 patients without CA19-9 data.  #4: 40 patients without Ki67 data; | | | | | | |

| **Supplementary table 2 The siRNA and shRNA sequence used in this study** | |
| --- | --- |
| siRNA |  |
|  | sense（5'-3'） |
| Human |  |
| PRMT5-1# | GGACCUGAGAGAUGAUAUATT |
| PRMT5-2# | GGGCUCAUUUGCUGACAAUTT |
| cMyc-1# | CACCUAUGAACUUGUUUCATT |
| cMyc-2# | GCCGUAUUUCUACUGCGACTT |
| Mouse |  |
| Prmt5-1# | CCCUUAAUCAGGAAGAUAATT |
| Prmt5-2# | CUCCAGUACUUGGAAUACUTT |
| cmyc-1# | GACGAGACCUUCAUCAAGA |
| cmyc-2# | CCUGAAGCAGAUCAGCAACAA |
|  |  |
| shRNA |  |
| Human |  |
| PRMT5-1# | CCCATCCTCTTCCCTATTAAG |
| PRMT5-2# | GCCCAGTTTGAGATGCCTTAT |
| Mouse |  |
| Prmt5-1# | GCACAGTTTGAGATGCCTTAT |
| Prmt5-2# | CCAGAACATCTGTGTGCGTTT |
|  |  |

| **Supplementary table 3 qRT-PCR primers used in this study** | |
| --- | --- |
| Human |  |
| c-Myc-F | TCCCTCCACTCGGAAGGAC |
| c-Myc-R | CTGGTGCATTTTCGGTTGTTG |
| PRMT5-F | TGTGGTGGTGCCCTATGA |
| PRMT5-R | GTGAGGCTCCCCTTTCTT |
| GAPDH-F | GGAGCGAGATCCCTCCAAAAT |
| GAPDH-R | GGCTGTTGTCATACTTCTCATGG |
| Mouse |  |
| c-myc-F | ATGCCCCTCAACGTGAACTTC |
| c-myc-R | CGCAACATAGGATGGAGAGCA |
| Gapdh-F | AGGTCGGTGTGAACGGATTTG |
| Gapdh-R | TGTAGACCATGTAGTTGAGGTCA |
| Prmt5-F | CTGAATTGCGTCCCCGAAATA |
| Prmt5-R | AGGTTCCTGAATGAACTCCCT |
| H4R3me2s-1-F | AGTGAGTTGCTGAGCAATTT |
| H4R3me2s-1-R | TGAGTTATCTAGGAGCCCC |
| H4R3me2s-2-F | TTAGAGAGACGCCTGGCC |
| H4R3me2s-2-R | AGCGCTCGGCTGAACTGT |
| H4R3me2s-3-F | TTTATATTCCGGGGGTCTGCG |
| H4R3me2s-3-R | AAGCCCTGCCCTTCAGGA |

| **Supplementary table 4 Antibodies for western blot and IHC used in this study** | |
| --- | --- |
| Gene | Cat Number |
| PRMT5 | 18436-1-AP |
| c-MYC | 67447-1-Ig |
| Ki67 | 27309-1-AP |
| Cleaved PARP1 | 9451-s |
| H4R3me2s | A-3718-050 |
| Cyclin D1 | 2978 |
| Cleaved Caspase 3 | 9644 |
| Beta-actin | 4967 |
| Anti-Histone H4 (symmetric methyl R3) | AB5823 |
| Symmetric Di-Methyl Arginine Motif | 13222 |
